# Targeting Endothelin-1 Receptor/β-Arrestin-1 Axis in Ovarian Cancer: From Basic Research to a Therapeutic Approach

**DOI:** 10.3389/fendo.2019.00609

**Published:** 2019-09-04

**Authors:** Piera Tocci, Laura Rosanò, Anna Bagnato

**Affiliations:** ^1^Preclinical Models and New Therapeutic Agents Unit, Istituto di Ricovero e Cura a Carattere Scientifico (IRCCS), Regina Elena National Cancer Institute, Rome, Italy; ^2^Institute of Molecular Biology and Pathology, CNR, Rome, Italy

**Keywords:** endothelin-1, endothelin-1 receptors, ovarian cancer, β-arrestin-1, G-protein coupled receptors

## Abstract

Recent studies imply a key role of endothelin-1 receptor (ET-1R), belonging to the largest family of G protein-coupled receptors (GPCR), in the regulation of a plethora of processes involved in tumorigenesis and metastatic progression. β-arrestin-1 (β-arr1) system has been recognized as a critical hub controlling GPCR signaling network, directing the GPCR's biological outcomes. In ovarian cancer, ET-1R/β-arr1 axis enables cancer cells to engage several integrated signaling, and represents an actionable target for developing novel therapeutic approaches. Preclinical research studies demonstrate that ET-1R blockade by the approved dual ET_A_R/ET_B_R antagonist macitentan counteracts β-arr1-mediated signaling network, and hampers the dialogue among cancer cells and the tumor microenvironment, interfering with metastatic progression and drug response. In light of major developments in the ET-1R signaling paradigm, this review article discusses the emerging evidence of the dual ET-1R antagonist treatment in cancer, and outlines our challenge in preclinical studies warranting the repurposing of ET-1R antagonists for the design of more effective clinical trials based on combinatorial therapies to overcome, or prevent, the onset of drug resistance.

## Introduction

G protein-coupled receptors (GPCR) are major therapeutic targets because their signaling, influencing many cellular biochemical activities, phenotype plasticity, and gene expression, touches numerous aspects of human endocrinology, physiology, and pathophysiology, including tumorigenesis (1, 2). Among these, the endothelin-1 (ET-1) receptors (ET-1R), ET_A_ receptor (ET_A_R), and ET_B_ receptor (ET_B_R), exert critical functions in many tumor settings and their aberrant expression has been observed in several malignances including ovarian cancer (OC) (3). Surgery and platinum-based chemotherapy represent the standard approach for OC patients. Clinical evidences indicate that even after initial response, the majority of OC patients relapse and acquire chemotherapy resistance (4–6). The limited clinical response observed in OC patients may be related to the integration network of different pathways, including ET-1 signaling. ET-1R activation occurs through the agonist-dependent binding of three 21-amino acid isoforms, ET-1, ET-2, and ET-3 (7–9), and their eclectic effects are due to the different grades of affinity of ET_A_R and ET_B_R for the three ligands (7–12). Indeed, ET_A_R exhibit higher affinity for ET-1 and ET-2, while ET_B_R binds the three ETs isoforms with equal affinity (8).

ET-1R activation mechanism have long been reported as a process consisting of the ETs bound to the receptor and G protein coupling, which triggers a signaling cascade including an increase in intracellular Ca^2^, activation of Rho-kinase, or adenylate cyclase/cyclic adenosine monophosphate pathway, and the transactivation of the epidermal growth factor receptor (EGFR) (3, 13, 14).

In addition to activating the G-protein pathway, ET-1R activate the β-arrestins (β-arr) pathway, known as β-arrestin-1 (β-arr1) and β-arrestin-2 (β-arr2), which guiding ET-1R internalization by the engagement of clathrin-coated pits, directs ET-1R recycling or lysosomal degradation, precluding G-protein coupling even in presence of a persistent ET-1 stimulus (15–18). While both ET-1R are internalized by β-arr and clathrin-dependent mechanisms, ET_A_R are recycled to the cell membrane while ET_B_R are lysosomal degraded (19). Interestingly, increasing evidence points out the existence of a signaling concept by which ET-1R reinforces and prolongs its signaling pattern through β-arr1, which favoring multi-protein complex formation, defines new signaling outputs parallel to the traditional G-protein-dependent one (3, 20). In particular, β-arr1 converts protein-protein interaction into a connected signaling network counting several oncogenic pathways, in both the cytoplasm and the nucleus (3, 20).

Downstream of ET-1R, the recognition of this β-arr1 role may be particularly relevant for the design of novel therapeutic combinations able to efficiently hamper also the ET-1R/β-arr1-dependent branch, besides the G-protein-mediated routes (3). In this scenario, this review aims to portray the multiple facet of ET-1-dependent signaling, describing the impact of β-arr1 activity on the integration between ET-1R signaling with other regulatory pathways which may influence both the tumor and the surrounded TME. The availability of appropriate preclinical models will allow more reliable studies in physio-pathological complex context to validate more effective therapeutic approaches able to antagonize the ET-1R/β-arr1 signaling network in OC and other malignancies.

## The Role of ET-1R/β-arr1-Mediated Functions in Ovarian Cancer

### Integrated Network of ET-1R/β-arr1-Mediated Signaling and Other Pathways

The integration of diverse signaling pathways operating concurrently within tumor cells may represent a mechanism to escape drug response. In this view, the identification of interconnected pathways, as critical vulnerabilities that allow OC progression and chemotherapy resistance, represents an essential tool to design promising therapeutic combination against OC. An increasing body of evidence has well-documented the close connection between ET-1/ET-1R axis and other signaling pathways in cancer. Indeed, β-arr1 may influence ET-1R signaling, physically complexing with multiple signal transducers, which exhibit different functions and localization, including cytoplasmic and nuclear proteins (3, 21–24), as well as cytoskeletal components (25–27), therefore promoting the integration of ET-1R signaling with different intracellular pathways. By acting as hub able to engage a pool of oncogenic intermediates, β-arr1 controls in a fine-tuning manner several aspects of ET-1-induced functions related to OC progression, including cell growth, cell survival, invasion, and chemoresistance (3). An example of how β-arr1, mediating the cross-talk of GPCR with other receptors, fosters ET-1R signaling network sophistication is represented by the crosstalk between ET-1R and the receptor tyrosine kinase (RTK) family members, as EGFR or the vascular endothelial growth factor receptor-3 (VEGFR-3) (28), through the recruitment of SRC, that promotes the transactivation of RTK in OC cells (3, 28).

Recent studies reported that β-arr1 signaling, converging on cytoskeleton remodeling-related signaling routes, generates dynamic morphological changes which produce the force required for changes in cell shape leading to cell invasion (29). In this regard, it has been demonstrated that downstream of ET-1R, β-arr1 activates a signaling cascade by interacting with PDZ-RhoGEF which, in turn, induces RhoC GTPase and cofilin pathway, and the formation of actin-rich invasive protrusions named invadopodia (25). β-arr1-associated molecular complexes during invadopodia maturation require the interaction with ENA/VASP family members, such as hMENA that regulates the cytoskeleton dynamic behavior of different cell types (26). The activation of ET-1R promotes the formation of a ternary complex consisting of β-arr1/hMENA/PDZ-RhoGEF which induces RhoC signaling, mediating extracellular matrix (ECM) degradation (26). More recently, it has been disclosed a new mechanistic association among ET-1R/β-arr1 axis and the integrin-related protein IQ-domain GTPase-activating protein 1 (IQGAP1) that participates to cytoskeleton remodeling, and invadopodia-dependent ECM degradation (27). In particular, β-arr1/IQGAP1 complex induces Rac1 inhibition and a concomitant RhoA and RhoC activation, indicating that ET-1R-driven β-arr1 interactions regulate the specific inputs for invadopodia formation and activation (27). This aspect further updates the landscape of the ET-1R/β-arr1-integrated pathways and may magnify the understanding of the specific β-arr1 contribution to such network complexity.

A noteworthy β-arr1-mediated cross-talk is that between ET-1R axis and β-catenin signaling. ET-1R activation induces β-arr1 recruitment at the cell membrane, where β-arr1 may trigger β-catenin stabilization through two modalities: the first one promoting the EGFR transactivation via SCR, β-catenin tyrosine phosphorylation and in turn its activation; the second one by inhibiting the β-catenin destruction complex, constituted by glycogen synthase kinase-3β (GSK-3β), axis inhibition 1 (AXIN1), adenomatous polyposis coli (APC), and β-transducin repeat containing protein (β-TrCP), therefore promoting β-catenin activation (30). Of note, the β-arr1-dependent ET-1R/β-catenin signaling integration is not limited to the cytoplasm. Indeed, this signaling interplay culminates into the nucleus where β-arr1/β-catenin physical interaction leads to β-catenin nuclear translocation, p300 recruitment on β-catenin target genes promoters, favoring chromatin acetylation and transcriptional activity of β-catenin/T-cell factor 4 (TCF4) that promotes gene expression, including *EDN1* (ET-1 gene), fueling an autocrine loop that sustain a persistent β-catenin activation, that fosters chemoresistance and metastatic behavior (21, 22).

More recently, it has been reported that the nuclear binding of β-arr1 to the hypoxia-inducible factor-1α (HIF-1α) creates a new signaling channel that connects ET-1R pathway to HIF-1α activity. In OC cells, β-arr1, as a nuclear co-factor, directs the recruitment of HIF-1α, as well as of p300, on hypoxia responsive elements contained within HIF-1α target gene promoters, promoting the transcription of pro-angiogenic genes, such as *EDN1* and *VEGF* (23). Consistent with these results, a ChIP-Seq analysis in prostate cancer cells exposed to pseudo-hypoxic conditions highlights the partial overlay of binding sites of β-arr1 and p300 within promoters and intronic regions. The identification of non-overlapping sites suggests that β-arr1 may regulate gene transcription also autonomously from p300 (31). In addition, the analysis of both β-arr1 and HIF-1α transcriptomes in breast cancer shows the overlap of the two gene profiles, including known HIF-1α targets such as those required in neovascularization, and aerobic glycolysis (32).

Similarly, it has been reported that β-arr1 allows the interfacing of ET-1R axis with the nuclear factor κB (NF-κB) signaling in OC cells. In particular, ET-1R activation, in a β-arr1-dependent manner, induces the phosphorylation of p65 leading to its activation and in parallel IκB-α phosphorylation, inducing its degradation. These two steps are critical for the β-arr1-dependent p65 nuclear accumulation and transcriptional activity. The existence of the cross-talk between ET-1R/β-arr1 and NF-κB provide a further attempt of how ET-1R/β-arr1 axis may affect chromatin remodeling and gene transcription regulation (24).

Regarding the ability of β-arr1 to interconnect oncogenic signaling pathways, a recent study discloses the interplay between ET-1R/β-arr1 axis and the Hippo transducers YAP and TAZ in patient-derived high-grade serous ovarian cancer (HG-SOC) cells and in breast cancer cell lines carrying *TP53* mutations (mutp53) (33), indicating a therapeutic option for the treatment of mutp53 cancers and opening new prospects on the regulation of YAP/TAZ biology (34, 35). Mechanistically, β-arr1 engages a physical interaction with the two related transcription co-activators YAP and TAZ into the cytoplasm where mediates its de-phosphorylation, leading to YAP/TAZ cytoplasmic-nuclear shuttling and activation in a G-protein independent manner. In parallel, β-arr1 interacting with a RhoGEF family member, Trio, integrates an additional signaling route that includes RhoA GTPase, and actin cytoskeleton activity, further favoring YAP/TAZ nuclear accumulation. As co-transcriptional factor, YAP binds TEAD as critical oncogenic transcription factor (36). In the nucleus, β-arr1 enrolling mutp53 on YAP/TEAD target gene promoters, becomes part of a ternary complex consisting of β-arr1/YAP/mutp53 that induces the aberrant expression of TEAD target genes. Remarkably, this transcriptionally active complex mediates also *EDN1* transcription, thus magnifying a positive feed-back loop that sustains a persistent YAP activation, which induces cell proliferation, survival and invasion. Moreover, in breast cancer cells β-arr1 may anchor also NFY transcriptional factor together with YAP and mutp53 (33, 37), inducing the transcription of the proliferative genes, further expanding the repertoire of β-arr1 nuclear partners and proving that β-arr1/mutp53 cooperation may support the transcription of YAP-associated signature. Clinically relevant, the expression of ET-1, ET_A_R, and YAP were simultaneously up-regulated in HG-SOC tissues compared to normal ovarian tissues. Additionally, HG-SOC patients harboring *TP53* mutations, with a combined high expression levels of ET-1R/β-arr1/YAP have a worse prognosis compared to patients who lack this network-based signature, emphasizing the poor outcomes generated by the integration between ET-1R/β-arr1 and YAP pathways and contribute to identify a predictive gene signature for recurrent HG-SOC (33).

These findings highlight the multimodality by which β-arr1, acting not only as a cytoplasmic signal transducer but, more significant, as a dynamic nuclear linker that guides the positioning of transcriptional factor and co-factors, regulates epigenetic control and, in turn, defines a highly characteristic transcriptional profile, creating an additional layer of ET-1R signaling regulation in oncogenic transcriptional activity (Table 1).

**Table 1:** Endothelin-1-induced β-arrestin-1 cooperation with transcription factors in cancer cells.

**Transcription factor**	**Biological role**	**Transcriptional effect**	**References**
β-catenin/TCF4	Induction of migration, invasion, epithelial-to-mesenchymal transition, chemoresistance, vascularization, intravasation, and metastatic progression in ovarian cancer cells	ACTIVATION	(21, 22)
HIF-1α	Promotion of pro-tumorigenic behavior in ovarian, prostate and breast cancer cells and induction of pro-angiogenic effects in endothelial cells	ACTIVATION	(23, 31, 32)
NFκB	Induction of cell survival in ovarian cancer cells	ACTIVATION	(24)
YAP/TEAD	Induction of cell proliferation, survival, and invasion in patient-derived high-grade serous ovarian cancer cells and breast cancer cells carrying *TP53* mutations	ACTIVATION	(33)
Mutant p53	Promotion of cell proliferation, survival, and invasion in patient-derived high-grade serous ovarian cancer cells and breast cancer cells carrying *TP53* mutations	ACTIVATION	(33)
NFY	Induction of cell proliferation in breast cancer cells carrying *TP53* mutation	ACTIVATION	(33)

### ET-1R/β-arr1-Mediated Cross-Talk With Other Signaling in the Tumor-Microenvironment

The interfacing of transformed cells with the surrounded tumor-associated elements from the TME, which include cancer-associated fibroblasts (CAF), endothelial cells (EC), and immune cells, as lymphocytes and tumor-associated macrophages (TAM), as well as the interaction between tumor cells and the ECM, impacts on cancer growth, progression and clinical outcome. A deep knowledge of how the dialogue among tumor cells, TME elements, and ECM may shape clinical response is an unmet medical need in oncology. Regarding this aspect, emerging data indicate that ET-1 signaling has a significant influence on several pathways and cellular processes involving both the tumor and the TME, thus emerging as a regulator of the signaling interchange between tumor and stromal compartment. In this perspective, it has been demonstrated that ET-1 is a regulator of tumor stroma. Indeed, tumor cells produce and secrete ET-1 which may facilitate tumor stroma remodeling (38, 39). In particular in many tumor type, including OC, it has been reported that ET-1 activating the ET-1R, both expressed by fibroblasts isolated by normal tissues near to cancer tissues, promote their growth, migration and contraction, as well as the production of ECM modifying factors (40), suggesting that such cross-talk may take place through the paracrine release of ET-1 that, fostering the formation of a prone tumor stroma, participates to create a niche that support tumor maintenance.

In addition, ET-1 seems to regulate also the immune environment at different levels, acting on different subclasses of immune effectors. Indeed, ET-1 appears to modulate dendritic cells (DC) behavior and activity (41). Moreover, a transcriptional profile conducted in OC shows that the over-expression of ET_B_R is negatively associated to the recruitment of tumor-infiltrating lymphocytes (TIL), which depends on the ET_B_R-dependent reduction of endothelial intercellular adhesion molecule 1 (ICAM1) expression. In line with this, ET_B_R interfering increases the adhesion of T cells to the endothelium, favoring the homing of T cells to the tumor (42–44). These observations indicate that ET-1R potentially may control the ongoing immune response in the TME.

As above reported, β-arr1 acts as an initial activator of the cross-talk between ET-1R and RTK, including the VEGFR-3 (3). In addition, both ET_B_R and VEGFR-3, as well as their associated ligands, ET-1, VEGF-A, VEGF-C and -D, act as environmental regulators affecting the blood and lymphatic endothelial cells (LEC) behavior (45, 46). In detail, it has been proved that ET-1, mediating ET_B_R activation and cooperating with hypoxia, induces the release of pro-angiogenic and lymphangiogenic factors, as VEGF-A -C -D expression and, in turn promotes EC and LEC growth and invasion inducing neo-angiogenesis and lymphangiogenesis (46). More relevant, the dual capacity of ET-1/VEGF inter-relation of simultaneously influencing the tumor and the TME emerges also in their ability to control the mutual regulation between tumor cells, EC, LEC, and hypoxia, directing at the same time tumor aggressiveness and angiogenic activities. In the tumor, hypoxia induces ET-1 expression, along with VEGF-A and -C release, through HIF-1α and HIF-2α (3, 45, 46). The autocrine/paracrine interchange of these angiogenic factors, promotes tumor progression and morphological changes in EC and LEC, revealing that such signaling interplay sustains tumor development in a permissive TME. Considering that tumor cells, EC and LEC express ET_B_R and VEGFR-3 as well as their cognate ligands, and taking into account that β-arr1 may coordinate the cooperation between ET_B_R- and VEGFR-3-related pathways (46), it's tempting to speculate that β-arr1 may actively take part also to the ET-1/VEGF-induced bidirectional communications between LEC, EC, and tumor cells. The above hypothesis may be further supported by a novel mechanism by which β-arr1, directly regulating VEGFR-3 signaling and expression in human microvascular EC from lung, favors the development of pulmonary arterial hypertension (PAH) (47). Indeed, it has been reported that β-arr1 interferes with VEGFR-3 internalization and degradation, promoting its signaling. In line with this, β-arr1 knockout mice develop acute PAH that is related to the loss of VEGFR-3 signaling (47).

For all the above mentioned findings, we can consider ET-1R/β-arr1 system as a complex signaling machinery for the tumor/endothelial/immune cells input interchange in the tumor milieu, sustaining angiogenesis, lymphangiogenesis, and immune system control, thus providing an advantage to cancer cells to growth and metastasize.

## Interfering With ET-1/β-arr1 Signaling Network for New Therapeutic Options in Ovarian Cancer

**Figure 1:**
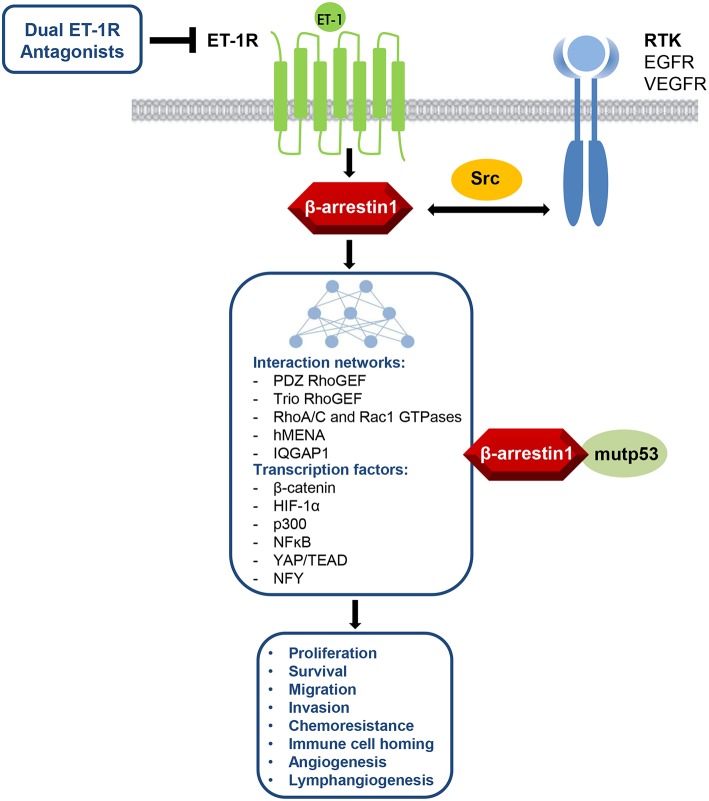
ET-1R/β-arr1 acts as signaling hub regulating several cancer hallmarks via its interaction networks between GPCR and RTK pathways with different cytosolic, cytoskeletal, or nuclear signaling nodes, including also the control of mutant *TP53* (mutp53) oncogenic activity. This knowledge makes ET-1R/β-arr1 an attractive target for therapeutic intervention by using dual ET-1R antagonists.

In this review, an array of pathophysiological roles for the β-arr1-mediated pathways upon ET-1R activation in ovarian cancer is described (Figure 1). Thus, ET_A_R/β-arr1 axis transmits signals to the nucleus, fostering early and late steps essential to metastatic progression and drug resistance. Therefore, besides the complexity of G-protein-mediated signaling (48), the disruption of the ET_A_R/β-arr1 interaction can impair several hallmarks of cancer, representing a possible avenue for therapeutic intervention. The biased agonist that can transduce ET-1 signaling either through G-protein or β-arr1 leads to the new paradigm of signal-biased antagonists (49–51). The therapeutic strategy to block ET-1 activities has evolved in the clinic, mainly for PAH, by using orally active small molecule antagonists, targeting selectively ET_A_R or both ET_A_R and ET_B_R. Besides the small molecules, other chemical compounds have been developed to target ET-1R, including monoclonal antibody antagonists and selective peptide agonists and antagonists (51). Different human cancers featured the benefit of targeting both ET_A_R and ET_B_R, in which ET-1R blockade with the dual ET_A_R/ET_B_R small molecule antagonists represents a suitable therapeutic option for ET-1R-expressing tumors. Indeed, the dual ET-1R antagonist macitentan concurrently attack tumor cells, which mainly express ET_A_R, and TME elements, that express ET_B_R, increasing anti-tumor immune and anti-angiogenic effects. In OC, silencing of β-arr1 or macitentan treatment, interfering with the interconnected pathways, inhibits tumor growth, angiogenesis (23), invasion, intravasation, and metastatic behavior (25–27), and sensitizes to platinum-based therapies (3, 22, 23). Additionally, macitentan is able to sensitize tumor cells to different cytotoxic and targeted agents in various preclinical tumor models, including colorectal cancer, glioblastoma, multiple myeloma, breast, and lung brain metastasis (3, 52–56). Future studies will guide to discover new biased antagonist with improved clinical profile. In this regard, the expected results of a phase III study (57, 58), that will explore the use of the long-acting (half-life of ≥12 h) aprocitentan, the active metabolite of the macitentan, in treatment-resistant hypertension, might indicate that a new licensed agent can be drug repurposed in oncology.

Since β-arr-1-mediated network might take place concomitantly in EC, LEC, fibroblasts, or in immune cells contributing to promote tumor progression in a suitable metastatic niche, future studies should also include reliable models that can recapitulate the complex TME. Therefore, the development of a platform of patient derived-preclinical models, including primary cell cultures, co-cultures with stromal elements, 3D organoids/tumoroids and patient-derived xenografts (PDX) is needed. These preclinical models, in particular those able to mimic the complexity of the TME landscape, as tumoroids (59), could be pivotal for modeling primary human tumor *ex vivo* and for drug screening. Considering the cancer vulnerability of ET-1R system and the mechanisms by which ET-1R antagonists synergize with other compounds (22, 52–56), further exploitation of the potential therapeutic paradigm of ET-1R blockade in combinatorial approaches in cancer now merits clinical consideration.

## Conclusions

The ET-1R/β-arr1 axis integrates signaling pathways related to several hallmarks of cancer, entailing tumor cells and TME elements, thus contributing to tumor growth, metastasis formation and drug response. Therefore, further insights on the role of ET-1R/β-arr1 axis may favor the development of novel effective therapies. In this context, the analysis of ET-1R/β-arr1 expression and β-arr1 interactome in OC and in different cancer types might be explored. The ability of β-arr1 to orchestrate the signaling network activated by ET-1/ET_A_R pathway has been revealed especially in the context of drug resistance that requires the inception of escape pathways through the interplay with RTK and other oncogenic nodes. In this context, the blockade of ET-1R/β-arr1 axis, impairing different signaling cascades overcomes compensatory mechanism of chemotherapy escape. Considering the complexity of ET-1R/β-arr1-driven signaling networks, further studies should elucidate whether combinatorial targeted approaches using dual ET-1R antagonists with other chemotherapeutic or targeted agents, would enable overcoming drug resistance. These studies should also advance our understanding of how ET-1R/β-arr1 related functions are integrated in specific tumor and TME cell types cooperating with oncogenic drivers or enabling signaling networks to potentiate tumor progression, metastasis, and drug response.

Additionally, the discovery of new biomarkers predictive of drug response will allow patient selection and will help in defining more effective combined treatments. In future multidisciplinary studies, the integration of genomic, transcriptomic, proteomic, metabolomic data, taking into consideration β-arr1 as crucial member of GPCR-mediated pathways, would provide a more detailed understanding of downstream signaling in cancer, which would facilitate the design of new effective combination therapies.

## Author Contributions

PT, LR, and AB conceived the study, participated in its design and coordination, and drafted the manuscript. All authors read and approved the final manuscript.

### Conflict of Interest Statement

The authors declare that the research was conducted in the absence of any commercial or financial relationships that could be construed as a potential conflict of interest.
